# Bridging Mental Health and Public Health

**Published:** 2009-12-15

**Authors:** Benjamin G. Druss, David Satcher

**Affiliations:** Rollins School of Public Health, Emory University; Morehouse School of Medicine, Atlanta, Georgia

A decade ago, the Surgeon General's office released its first report on mental health (1), calling for the full integration of mental health into the nation's public health system. The report synthesized the scientific literature on mental illness, concluding that mental disorders are among the most prevalent and costly conditions and that effective treatments can reduce their prevalence and decrease their adverse effect on other health conditions. The report took a broad public health approach, focusing not only on clinical diagnosis and treatment of mental illness but also on surveillance, prevention, and promotion of mental health (2).

The Surgeon General's report described research developments from the 1990s, the "Decade of the Brain," that helped establish the biological underpinnings of mental disorders and move mental health into the mainstream of research and specialty practice. The subsequent decade saw a dramatic rise in the proportion of the US population receiving mental health care (3) and a shift in the locus of treatment for mental illness away from specialty settings and toward primary care (4). During the same period, strategies for moving medical and psychiatric treatment from research into routine practice settings were developed and disseminated. In particular, research showed that integrated approaches could improve quality and outcomes of care in clinical settings on the interface of primary care and mental health (5). This research laid the groundwork for a broader strategy to integrate mental health and public health at a population level. The passage of the Paul Wellstone and Pete Domenici Mental Health Parity and Addiction Equity Act of 2008 was a step toward this goal.

As the lead government agency for the nation's public health, the Centers for Disease Control and Prevention (CDC) can play a central role in these efforts to integrate mental health and public health. Articles in this issue of *Preventing Chronic Disease* were developed by an expert panel convened by CDC's Division of Adult and Community Health on behalf of the National Center for Chronic Disease Prevention and Health Promotion (NCCDPHP). The panel was charged with examining how mental health should fit within NCCDPHP's mission. The articles provide the background for the panel's recommendations and cover a spectrum of public health activities, including surveillance, prevention and promotion, and the system and policy context for these proposed changes.

Freeman et al (6) provide a mixed report on the mental health surveillance systems available in the United States. Existing systems offer data about the prevalence and severity of mental disorders in the United States and their relationship to other chronic diseases and health behaviors, but they are limited by differences in methods, priorities, and lack of input from end users. Better coordination across and in the federal agencies that field these surveys could improve the value of the information, reduce redundancy, and increase the use of the data by researchers and policy makers.

Whereas the medical model focuses on understanding and treating disease, public health approaches take a broader view of health that includes both sickness and well-being. Manderscheid et al (7) describe health and illness not as a single continuum, but as distinct states that can exist simultaneously. This premise is compatible with the idea of recovery as a permanent condition that allows people to live fulfilling lives despite ongoing mental or physical symptoms. The recovery model has become central to the mental health advocacy and policy communities and was a guiding principle for the recommendations in the President's New Freedom Commission report (8). Recovery promotes a strength-based, public health approach that could easily be expanded to many people with chronic medical conditions.

Although public health activities must include surveillance and interventions across large populations, they must also account for differences across regional and cultural subgroups. The supplement to the Surgeon General's report, *Culture, Race, and Ethnicity*, noted that even greater racial and ethnic disparities exist for mental health care than for other types of health services (9) and that reducing these disparities will require close attention to issues of racial and cultural diversity. Primm et al (10) note that these disparities result not only from bias but also from social factors such as disadvantages in housing and income. Fully resolving these disparities will therefore require expanding beyond the formal health system and understanding the social determinants of mental health and well-being.

Given the decentralized and complex nature of mental health care, improvements must rely on partnerships at multiple levels. Primary care providers need to work more closely with mental health centers to ensure coordinated treatment (11). Counties need to develop relationships between mental health, medical, and local public health agencies (12). State public health agencies need to work more closely with mental health agencies (13). Finally, federal agencies need to better coordinate their efforts (14). In particular, CDC and the Substance Abuse and Mental Health Services Administration (SAMHSA), which have historically functioned in parallel but unconnected tracks, are now collaborating more closely on activities, such as jointly funding the surveillance of mental health and mental illnesses. Leaders from SAMHSA's Center for Mental Health Services were represented on the expert panel and are advising NCCDPHP on integrating mental health into its mission.

The nation is now poised to take the next step toward realizing the vision of integrating mental health and public health described a decade ago in the Surgeon General's report. Spiraling health care costs and the rising number of uninsured Americans have built momentum for health care reform, and it is clear that a population-based, public health approach — one that encompasses mental health — will be needed as a foundation for that reform (15).
